# Exercise Induced Left Bundle Branch Block Treated with Cardiac Rehabilitation: A Case Report and a Review of the Literature

**DOI:** 10.1155/2014/204805

**Published:** 2014-02-06

**Authors:** Nathan S. Anderson, Alexies Ramirez, Ahmad Slim, Jamil Malik

**Affiliations:** Cardiology Service, Brooke Army Medical Center, 3551 Roger Brooke Drive, San Antonio, TX 78234-6200, USA

## Abstract

Exercise induced bundle branch block is a rare observation in exercise testing, accounting for 0.5 percent of exercise tests. The best treatment of this condition and its association with coronary disease remain unclear. We describe a case associated with normal coronary arteries which was successfully treated with exercise training. While this treatment has been used previously, our case has a longer followup than previously reported and demonstrates that the treatment is not durable in the absence of continued exercise.

## 1. Introduction

**Figure 1:**
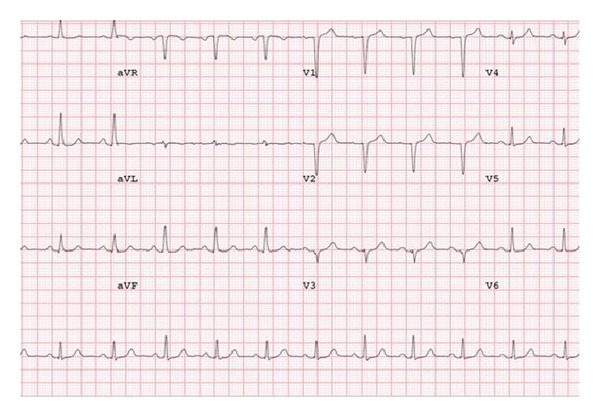
Baseline electrocardiogram demonstrating normal baseline conduction.

The patient was a 42-year-old woman who presented with exertional chest pain, palpitations, and dyspnea that resolved with rest. She had a normal physical exam and her only medication was an oral contraceptive. 12-lead electrocardiogram was normal with the following intervals: PR interval was 154 millisecond (msec), QRS was narrow at 82 msec, and QT interval was normal at 392 msec, corrected QT (QTc) using Bazett's formula was 431 msec (Figure 1). Laboratory tests including hemoglobin and cardiac troponin T were normal.

**Figure 2:**
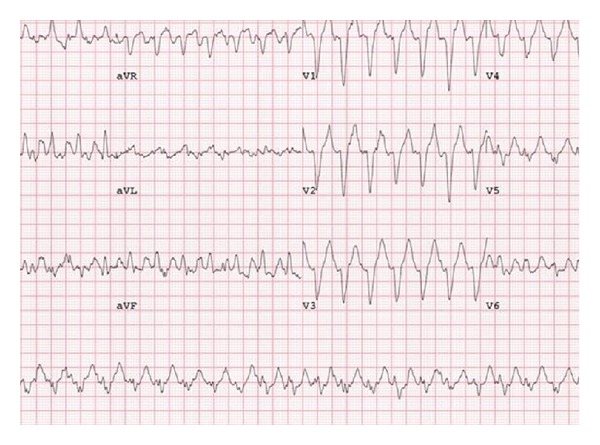
Electrocardiogram at peak exertion demonstrating left bundle branch block morphology.

She was referred for exercise stress testing using the Bruce protocol during which she developed a left bundle branch block (LBBB) with a QRS duration of 120 msec at a heart rate of 112 beats per minute (bpm) (Figure 2). During the aberrant conduction and at peak exercise, her symptoms of chest pain and palpitations returned. She was able to exercise through her discomfort, reaching a peak heart rate of 171 bpm and 10.4 metabolic equivalent (MET) at 9 : 11 min of exercise. The test was stopped due to limiting chest discomfort that persisted until her heart rate returned to 100 bpm at 2 : 30 min of recovery and normal conduction was restored. An echocardiogram was performed and revealed no structural abnormalities other than a small patent foramen ovale (PFO). Concerns regarding ischemia as the etiology for her conduction abnormalities prompted coronary angiography that demonstrated normal coronary arteries with no evidence of atherosclerosis.

The patient was a military service member on active duty status, which would require passing a physical fitness test, something her symptoms had not permitted. In the absence of structural heart disease leading to her conduction abnormality at peak exercise, patient was prescribed an exercise program in an attempt to improve symptoms with physiologic conditioning and left ventricular remodeling. Patient underwent cardiac rehabilitation exercise prescription with five times weekly 30-minute submaximal aerobic exercise. As previously reported by Heinsimer et al. [1], cardiac rehabilitation exercise training has been used to treat rate-related left bundle branch block with noted improvement in symptoms.

**Figure 3:**
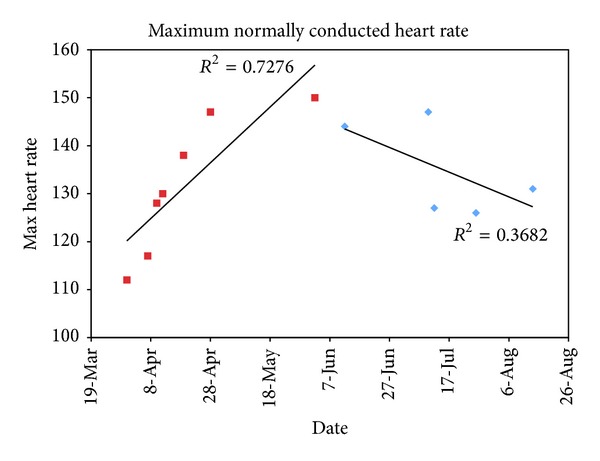
Plot of maximum normally conducted heart rates over time, demonstrating an increase in maximum rate with regular exercise training (red) and a decrease with nonadherence to regular training (blue).

After three months of regular exercise training with 30-minute sessions per day for five days a week, the patient's symptoms improved with development of LBBB and chest pain at a considerably higher heart rate of 150 bpm (Figure 3). The morphology of the LBBB remained the same. Notably, offset of aberrancy remained unchanged, with her last stress test demonstrating return to normal conduction at 108 bpm. With her symptoms improving, she became much less consistent in her attendance at cardiac rehabilitation sessions. As she became more noncompliant with her attendance, her heart rate threshold for development of symptoms with aberrancy dropped again to 120–130 bpm, a marginal improvement from her baseline.

## 2. Discussion

Symptomatic exertional rate-related left bundle branch block associated with symptoms of chest pain and palpitation was first described by Eichert in 1946 with subsequent reports by multiple other authors [1–5]. However, the prognosis and best treatment course have not been well established. Seven small case series have reported populations of rate-related left bundle branch block, and two have attempted to provide prognostic data [2, 5–10].

Virtanen et al. reported a series of seven patients with exertional chest pain and exercise induced LBB, who in the process of evaluating the cause of chest pain were found to have normal coronary arteriogram despite the persistence of the exertional chest pain associated with LBBB at peak exercise [11]. On the other hand, in a different case series of 11 patients with left bundle branch block induced with exercise treadmill testing, drawn from 4100 consecutive exercise tests at their institution, the incidence of obstructive coronary disease was found to be 63% (7 out of 11 patients) on left heart catheterization. These results lead the authors to conclude that exercise induced LBBB is almost always associated with coronary disease [6].

In a different study aimed at identifying the heart rate parameters during exercise at which LBBB is induced, in 2,584 consecutive patients who underwent treadmill testing, the incidence of exercise induced LBBB was 1.1% (occurred at range of 60 to 163 bpm). Of these 28 patients, 19 (68%) had no obstructive coronary disease on subsequent catheterization. Authors further demonstrated the rate at which the LBBB developed was important for determining prognosis. None of the patients in this study with exercise induced LBBB at heart rates over 125 bpm had coronary disease [5].

Williams et al. [8] published a case control series of 70 patients with exercise induced LBBB, drawn from a series of 17,277 consecutive treadmill tests. The control patients were matched according to the variables of sex, hypertension, diabetes, smoking, beta-blocker use, and history of coronary disease. Not every patient in this cohort underwent coronary angiography, but outcomes were followed for a mean of 3.7 years. A composite endpoint of all-cause mortality, revascularization (percutaneous or surgical), nonfatal myocardial infarction, or need for an implanted pacemaker or defibrillator was used. At four years of followup, the composite endpoint occurred in 10% of the control cohort, and in 19% of the case cohort. Significantly, this endpoint was independent of documented coronary disease, with an adjusted relative risk of 2.73 (see Table 1). Note that not every patient underwent diagnostic angiography.

The above studies demonstrate that exercise induced LBBB is a rare condition, occurring in less than one percent of exercise treadmill tests. The incidence of coronary disease in this population remains unclear. There exist no trials of therapy for these patients, and, despite the fact that several authors [1, 3, 10, 13] describe significant symptoms with this condition, discussions of treatment are limited to case reports. Pharmacologic therapy has been discussed, with nitroglycerin administration terminating the aberrant conduction in one patient [4]. Beta-blockers have been used to decrease the heart rate response to exercise and therefore avoid the development of the aberrancy. But only one study to date has demonstrated nonpharmacologic therapy through exercise training [2]. This study formed the basis of our treatment plan; however, we report a longer period of followup, which demonstrated that continued exercise is necessary to maintain the beneficial effects.

Vasey et al. [6] have proposed a mechanism for this aberrant conduction. The primary cause is delayed recovery, with one bundle branch having a block in phase 3 of the action potential, which can vary in length. With the increase in heart rate associated with exercise, eventually stimuli arrive from the proximal portion of the conduction system before the fascicle has repolarized and block occurs. This is generally coupled, in their model, with phase 4 hypopolarization, which causes bradycardia related LBBB; however, exploring this possibility would require an electrophysiological study, which was not performed in our patient. Critical to relating this experimental finding to our patient, however, is the observation that exercise induces upregulation of the potassium channels responsible for phase 3 of the action potential, with concomitant shortening of this phase. It seems reasonable to assume therefore that exercise training allows a shortening of phase 3 of the action potential and the increase in rate at which LBBB occurs. Deconditioning could reasonably be assumed to have the opposite effect, with the associated decrease in critical rate noted in our patient. It is worth noting that in one previous study, repeat testing years after the first evidence of exercise induced LBBB showed patients were developing the condition at lower heart rates and presumably developing symptoms with less activity [2].

In either case, treating the symptoms, allowing the patient to participate in more strenuous exercise, should have a morbidity benefit, as well as the mortality benefit which accrues from aerobic exercise.

## 3. Summary

Exercise-induced bundle branch block in the absence of coronary disease remains a rare condition in the United States. Though no treatment has yet been demonstrated to be effective in reducing mortality, treatment of this condition through the relatively simple intervention of cardiac rehabilitation proved an effective intervention to decrease symptoms. With regular exercise training, our patient was able to increase the rate at which she developed aberrant conduction and symptoms of chest pain and palpitations.

## Figures and Tables

**Table 1:** All published case series of patients with exercise induced LBBB, with incidence and prevalence of coronary artery disease.

	Authors/date							
	Virtanen et al., 1982 [11]	Wayne et al., 1983 [7]	Vasey et al., 1985 [6]	Heinsimer et al., 1987 [10]	Williams et al., 1988 [8]	Moran et al., 1992 [12]	Hertzeanu et al., 1992 [3]	Grady et al., 1998 [9]
Number of patients	7	11	28	15	37	29	11	70
Mean age in years	44.6	57	53	52	61	63	48	68
Normal perfusion imaging	Not reported	Not reported	Not reported	Not reported	Not reported	17	Not reported	Not reported
Abnormal perfusion imaging	Not reported	Not reported	Not reported	Not reported	Not reported	20	Not reported	Not reported
Normal coronary angiography	7	4	19	7	11	4	7	8
Abnormal coronary angiography	0	7	9	8	26	14	3	35
HR onset with no associated CAD	106 ± 30	94 ± 34	Not reported	124 ± 15	118	129 ± 32	85 ± 25	Not reported
HR onset with associated CAD	Not reported	104 ± 47	Not reported	124 ± 22	Not reported	114 ± 29	126 ± 25	Not reported
